# Hysterical Aphonia

**Published:** 1891-01-10

**Authors:** 


### HYSTERICAL APHONIA.

Hysterical aphonia, according to Dr. E. Fletcher Ingals, of Chicago, and others, depends upon paralysis of the lateral crico-arytenoid muscles. The author named above has an article on the subject in a recent number of the *Journal of the Medical Association of Chicago*. He states, it is a singular fact in accordance with what might be termed a conservative law of pathological selection, that in nerve trunks, not larger than a small needle, only those fibres are involved which supply the adductor muscles, while adjoining fibres supplying the abductor muscles are not implicated. Hysterical aphonia is usually suddenly developed, patients waking in the morning to find the power of speech lost. On inspection, the larynx has usually a normal appearance, but sometimes congestion is present. This form of aphonia is generally associated with some other manifestation of hysteria. It must not, however, be forgotten that aphonia sometimes follows violent inflammation of the larynx. It is also occasionally the result of lead, or arsenic poisoning. Gerhardt believes that it is sometimes of rheumatic origin. Many kinds of treatment have been adopted for hysterical aphonia with indifferent success. Occasionally the mere examination with a largyngoscope is successful, when the patient is very impressionable. Stimulating sprays and powders are sometimes beneficial. Electrical stimulation is not advantageous in protracted cases. But we fully agree with Dr. Ingals, when he says, "the most important part of the treatment consists in securing proper hygienic surroundings and diet, and the administration of tonics." Of tonics strychnine appears to be the most valuable. This remedy should be given at first in small doses, but the quantity should be steadily increased until physiological effects appear. The patients should be told what they are taking, and what physiological effects may result. In hysterical aphonia there seems to be a tolerance of this remedy. Dr. Ingals has given from one-tenth to one-eighth of a grain thrice daily, before the slightest constitutional effect could be detected, and he has no fear of so-called cumulative effects. As soon as twitching, or stiffness of the muscles, or intense nervousness is noted, the dose should be reduced. Several cases thus successfully treated are detailed by the author. Although hysterical aphonia cannot be regarded as dangerous, *per se*, still it may be connected with diseased conditions of the system which may prove dangerous. Sir M. Mackenzie stated some time ago, that aphonia of a purely functional nature occurs in the earlier stages of phthisis. And this fact should lead to careful examination of the lungs of every patient presenting, suffering from loss of voice, and especially when the patient is a young female. It may, however, be recollected that loss of voice may happen during the progress of phthisis, from organic change of structure. Notwithstanding all the author advances with regard to the efficacy of the treatment recommended for hysterical aphonia, we think that, as with other manifestations of hysteria, there is no certain remedy. As before mentioned, it often presents suddenly, and it frequently disappears as suddenly. And often the last remedy used is erroneously credited as the curative agent.

